# Pharmacists’ Behavioral Changes after Attending a Multi-Prefectural Palliative Care Education Program

**DOI:** 10.3390/pharmacy12030087

**Published:** 2024-06-04

**Authors:** Masahiro Yamada, Mayako Uchida, Masao Hada, Haruka Wakabayashi, Daigo Inma, Shunji Ariyoshi, Hidetoshi Kamimura, Tohru Haraguchi

**Affiliations:** 1 Department of Pharmacy, Kitakyushu Municipal Medical Center, 2-1-1, Bashaku, Kokurakita, Kitakyushu 802-0077, Japan; iryoyaku@aioros.ocn.ne.jp; 2 Department of Education and Research Center for Pharmacy Practice, Faculty of Pharmaceutical Sciences, Doshisha Women’s College of Liberal Arts, Kyotanabe 610-0395, Japan; yk19115@dwc.doshisha.ac.jp; 3 Department of Pharmacy, Japan Community Health care Organization Nankai Medical Center, 7-8, Tokiwanishimachi, Saiki 876-0857, Japan; hada-masao@nankai.jcho.go.jp; 4 A Public Interest Incorporated Foundation, Fukuoka Pharmaceutical Association, 2-20-15, Sumiyoshi, Hakata, Fukuoka 812-0018, Japan; the.sandman.is.coming.1985@gmail.com (D.I.); phd89314sa@power.email.ne.jp (S.A.); kamisan@fukuoka-u.ac.jp (H.K.); gucchi0819@pharmaunion.co.jp (T.H.); 5 Department of Pharmacy, Fukuoka University Hospital, 7-45-1, Nanakuma, Jonan, Fukuoka 814-0180, Japan

**Keywords:** pharmacists’ behavioral change, cancer, palliative care, education program, multi-prefecture, transtheoretical model

## Abstract

Central to the pharmacist’s role in palliative care is symptom management through direct participation in patient care and the provision of optimal pharmacotherapy to support patient outcomes. Consequently, palliative care requires extensive knowledge and action for patients with cancer. Therefore, this study aimed to evaluate how pharmacists’ behavior changed after attending a palliative care educational program. We conducted a web-based questionnaire survey examining the behavior of pharmacists regarding palliative care before participating in the program, two months after participating in the program, and eight months after participating in the program to determine their behavior and changes over time. For all questions, scores were higher at two and eight months after attending the program than before attending the program (*p* < 0.05). In addition, no significant difference was observed between two and eight months after attending the program for any question (*p* = 0.504–1.000). The knowledge gained from the educational program was used to repeatedly intervene with patients with cancer in order to address the various symptoms they experienced and maintain their behavior. The proven effectiveness of this program serves as a stepping stone for nationwide rollout across Japan’s 47 prefectures.

## 1. Introduction

Specialized palliative care is associated with improved symptoms, better end-of-life quality, decreased medical costs, and higher satisfaction with family assessments [1,2]. Early and continuous intervention in palliative care contributes to the quality of life of patients and an improved prognosis [3]. The World Health Organization (WHO) [4] states that palliative care “can be applied in the early stages of the disease in combination with other therapies aimed at prolonging life, such as chemotherapy and radiation therapy”. However, some patients with cancer reported that their palliative care needs were unmet [1].

Palliative care must be multidisciplinary, and guidelines have been established regarding the role of pharmacists in palliative care [5]. Central to the pharmacist’s role in palliative care is symptom management through direct participation in patient care and the provision of optimal pharmacotherapy to support patient outcomes. It is necessary to use various analgesics depending on the patient’s pain status, and the type and dose of analgesics must be adjusted accordingly. Analgesics may also be used to treat the side effects associated with anticancer drug treatment. However, if the anticancer drug treatment is effective, analgesics may not be necessary. Therefore, palliative care requires extensive knowledge and action for patients with cancer. Team medical care is also important to provide optimal pharmacotherapy. If pharmacists implement these guidelines, this will have a positive impact on patient outcomes. Cancer is the leading cause of death in Japan, and the involvement of pharmacists in palliative care is important [6]. For example, medical narcotics are used to relieve physical pain. However, the consumption of medical narcotics in Japan is extremely low compared with that in developed countries, and the treatment of cancer pain is considered inadequate. In fact, pharmacists are hesitant to take proactive actions if they lack knowledge [7]. Another report stated that lack of training and clinical expertise is a barrier to the practice of evidence-based medicine [8]. Therefore, complementary training should be provided to pharmacists to enable them to be proactively involved in the palliative care of patients with cancer.

In a previous study, we developed an extensive and systematic educational program on palliative care for patients with cancer and found that it improved their palliative care knowledge [9,10]. However, it was unclear whether pharmacists were able to take action for patients with cancer to alleviate their symptoms based on the knowledge gained. Pharmacists must acquire knowledge and be able to provide better drug therapies to patients based on this knowledge. We set the learning goal of the educational program as “changing the behavior of pharmacists by inculcating knowledge as one of the competencies necessary for palliative care”. Therefore, in this study, we used a transtheoretical model to evaluate the behaviors of pharmacists who participated in a palliative care educational program. Very few studies have focused on pharmacists’ behavioral changes in palliative care.

## 2. Methods

### 2.1. Program

This program consists of a total of 24 items, with the aim of teaching pharmacists the “basic knowledge” they need to manage pain and medications, as well as the “multidisciplinary collaboration” needed to collaborate with other medical professionals. These 24 items are designed to be learned over six days (4 items per day), and sessions will be held 2–3 times a year over a period of approximately three years. Table 1 shows the contents of all 24 items and the occupations of the performers in the sessions that were conducted from May 2018 to March 2021.

### 2.2. Development and Verification of This Program

In the process of developing the educational program, we focused on being able to accurately assess patient pain and being able to treat gastrointestinal, respiratory, psychiatric, and urinary symptoms as a means of alleviating symptoms other than pain symptoms. Furthermore, we collaborated with doctors, nurses, nutritionists, and medical social workers.

The program was based on a textbook published by the Japanese Society of Palliative Medicine and Pharmaceutical Sciences, from which 24 items were extracted.

We verified and reported that after participating in this educational program, participants were able to accurately assess patient pain, alleviate various symptoms besides pain, and collaborate with multiple professionals [9,10].

### 2.3. Questionnaire Survey

A web-based questionnaire survey was conducted among pharmacists who participated in this program and resided in nine prefectures of southwestern Japan (Fukuoka, Saga, Nagasaki, Kumamoto, Oita, Miyazaki, Kagoshima, Okinawa, and Yamaguchi). Behaviors related to palliative care were analyzed at three time points: before taking part in the program (April 2018), two months after taking part in the program (May 2021), and eight months after taking part in the program (November 2021). First, the participants’ behavior before participating in the program and two months after participating in the program was surveyed from 10 May to 21 June 2021. Next, the participants’ behavior eight months after participating in the program was surveyed from 9 November to 14 December 2021. All the analyses were conducted anonymously. No compensation was offered to respondents for their participation. 

The survey items evaluated age, years of experience as a pharmacist, place of work, place of residence, and behaviors related to palliative care (12 questions). A consent form was received before the program began and the participants were asked to respond to a questionnaire. The survey was conducted online, and the participants self-reported their behaviors. 

Table 2 shows the questionnaire regarding behavioral changes related to palliative care. The participants were asked to self-rate at each time point and respond on a scale of 1 (not good at all) to 10 (good enough).

Q1–3 addressed whether specific actions were taken regarding pain symptoms in patients. Q4–7 addressed whether the participants could take specific actions regarding symptoms other than pain. Q8–12 addressed whether in-team medical care takes action in other occupations.

### 2.4. Transtheoretical Model

The transtheoretical model codifies the stages and process of behavior change [11,12]. Changes in behaviors involve progression through five stages: precontemplation, contemplation, preparation, action, and maintenance. Each stage is defined as follows: precontemplation means a person has no intention of changing the problem behavior within the next six months; contemplation means a person intends to change within the next six months; preparation means a person plans to change within the next month; action means a person has already changed their behavior over the six months, and maintenance means a person kept changing their behavior for more than six months.

We found it difficult to evaluate how long a behavior needs to continue, because there are few objective indicators to evaluate it. Therefore, we referred to the two concepts of “action” and “maintenance” in this model, such that we defined the behavior within 6 months from the first day of taking this program as the “behavioral period”, and the behavior that was maintained even 6 months after taking this program as that of the “maintenance period”. 

### 2.5. Statistical Analysis

To evaluate reliability, the split-half method was used. The reliability coefficient (ρ) was calculated from the Spearman–Brown coefficient to confirm internal consistency. Friedman’s test was used to determine whether there was a change in scores for each participant across the three time points, and Bonferroni’s adjustment [13] was used for group comparisons (post hoc analyses). The significance level was set at *p* < 0.05. SPSS statistics version 27.0 was used for all statistical analyses.

## 3. Results

### 3.1. Questionnaire Collection

A total of 365 participants participated in this study. Of these, 138 (37.8%) responded to the questionnaire survey two months after attending the program, and 142 (38.9%) responded eight months after attending the program. We analyzed the responses of the 96 (26.3%) participants who completed the survey both two and eight months after attending the program.

### 3.2. Respondents’ Characteristics

Table 3 presents the respondents’ age, years of experience as a pharmacist, place of work, and residence eight months after attending the program. The most common age group was participants in their 40s (34, 35.4%), followed by those in their 50s (33, 34.4%). There were zero participants in their 20s. Regarding years of experience as a pharmacist, 50 (52.1%) participants indicated “21 years or more”. By workplace, 75 participants (78.1%) worked at pharmacies, 20 (20.8%) at hospitals or clinics, and 1 (1.1%) at other hospitals. Most participants were from Fukuoka Prefecture (47, 49.0%), followed by Yamaguchi Prefecture (10, 10.4%), Saga Prefecture, Kumamoto Prefecture, and Oita Prefecture (8 each, 8.3%). 

### 3.3. Pharmacists’ Behavioral Changes for Each Question

**Figure 1:**
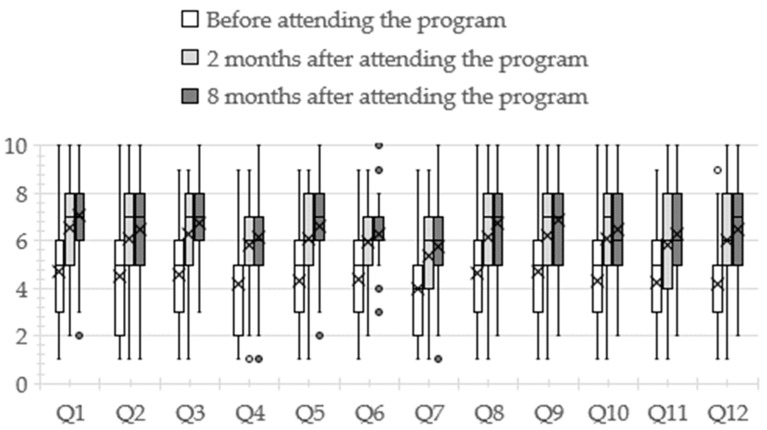
Behavioral changes of pharmacists who participated in the palliative care educational program. Small circles are outliers. An outlier is a value greater than “3rd quartile plus interquartile range times 1.5” or less than “1st quartile minus interquartile range times 1.5”. Crosses indicates the average value.

Figure 1 depicts the participants’ behavioral change scores for each question at all three time points. The questions are listed in Table 2. For all questions, scores were higher two and eight months after attending the program than before attending (*p* < 0.05). In addition, no significant difference was observed between two and eight months after attending the program for all questions (*p* = 0.504–1.000). The reliability coefficient ρ for each question ranged from 0.97 to 1.00 (Q1, 0.987; Q2, 0.996; Q3, 0.998; Q4, 0.996; Q5, 0.992; Q6, 0.988; Q7, 0.995; Q8, 1.000; Q9, 0.991; Q10, 0.991; Q11, 0.992; Q12, 0.977).

## 4. Discussion

In this study, changes in pharmacists’ behavior toward patients and healthcare professionals were observed, with scores on all 12 questions increasing at two and eight months after attending the palliative care program compared with before attending the program. This result is similar to that of our previous report on better behavioral changes among pharmacists in a single prefecture after attending a palliative care educational program for cancer [14]. The program proved effective even when extended to nine prefectures. The results of this study are supported by previous reports on behavioral changes in pharmacists after attending an evidence-based medicine-learning program [15]. Pharmacists who participated in a health promotion training program that included blood pressure control reported changes in their behavior and attitudes, indicating that training programs are needed to increase pharmacists’ confidence [16]. These findings suggest that pharmacists can act proactively toward patients and healthcare professionals based on the knowledge gained from our educational program. Continuing education is required to correct this learning gap in community pharmacies [17]. The continuation of this program is important for providing better palliative care to patients.

The WHO states that palliative care is multifaceted and focuses on approaches to improving the quality of life of patients with life-threatening illnesses and their families. Palliative care is aimed at “the early identification of pain and other physical, psychosocial, and spiritual problems and the prevention and alleviation of suffering through impeccable assessment and treatment” [18]. Guidelines from the American Society of Clinical Oncology recommend that patients with cancer receive palliative care when standard treatment is initiated [19]. Therefore, pharmacists’ role in palliative care is expected to become increasingly important. Pain is one of the most common symptoms experienced by patients with cancer [20]. Opioid analgesics are important drugs for pain treatment and pharmacists are responsible for alleviating pain in patients [21]. In addition to pain, patients with cancer experience unpleasant respiratory, psychosomatic, gastrointestinal, and urinary symptoms [22,23,24,25]. In palliative care, professional healthcare teams must include doctors, nurses, nutritionists, physical therapists, and medical social workers [26,27,28,29]. Therefore, pharmacists must fully understand the roles of other healthcare professionals and collaborate appropriately. Importantly, this study showed positive behavioral changes in patients’ pain and non-pain symptom relief (Q1–7). Although the level of medical resource availability differed by region, pharmacists in all nine prefectures deepened their understanding of the roles of multiple professionals and were able to put what they had learned into practice (Q8–12). 

This study found no change in pharmacists’ behavior scores at two and eight months after completing the program, and the scores remained relatively high. In other words, the score after two months was maintained for an additional six months. The repeated performance of a new behavior increases the likelihood that the behavior will become habitual and be maintained [30]. The results suggest that the knowledge gained from the educational program was used to repeatedly intervene with patients with cancer to address the various symptoms they experienced and that their behavior was maintained. According to the transtheoretical model, when a person changes their behavior or lifestyle, they undergo five stages: “pre-contemplation”, “contemplation”, “preparation”, “action”, and “maintenance” [31]. The maintenance phase is defined as a behavioral change that lasts for more than six months. As applied to the present results, the pharmacists’ behavior change after attending the educational program lasted for six months, implying that the pharmacists’ behavior improvement was not temporary. In general, most educational programs have some short-term effectiveness, and it is unlikely that learners will know less after the program ends than they did before. However, the value of this study is that the pharmacists used the knowledge they acquired to improve their behavior compared to that before participating in the educational program. Additionally, behavioral improvements were maintained over a long period of time up to 6 months after participation.

This study has some limitations, including the possible bias that the pharmacists who participated in this study were highly motivated because it was conducted retrospectively. Therefore, further prospective studies are required to obtain more accurate results. This study suggests that expanding palliative care programs would result in better behavioral changes among pharmacists. In addition, we found that pharmacists’ behavioral changes were maintained. We believe that this palliative care program can serve as a stepping stone and model for nationwide expansion as we were able to validate the program in multiple prefectures rather than in a single prefecture.

## Figures and Tables

**Table 1:** Items covered in the six days (a total of 24 items).

Days	Specific Items	Performers
Day 1		
Day 1-1	Palliative care in general	Pharmacist
Day 1-2	Pain management in general	Physician
Day 1-3	Infusion therapy at the end of life	Physician
Day 1-4	Role of physicians in charge of physical symptoms	Physician
Day 2		
Day 2-1	Characteristics of non-opioid analgesics	Pharmacist
Day 2-2	Management of psychiatric symptoms	Physician
Day 2-3	The role of the physician in charge of mental symptoms	Physician
Day 2-4	The role of the nurse	Nurse
Day 3		
Day 3-1	Characteristics of opioid analgesics	Pharmacist
Day 3-2	Side effects of opioid analgesics	Pharmacist
Day 3-3	Digestive symptoms at the end of life	Physician
Day 3-4	The role of the nutritionist	Nutritionist
Day 4		
Day 4-1	Analgesic aids	Pharmacist
Day 4-2	Dependence/tolerance of opioid analgesics	Pharmacist
Day 4-3	Respiratory symptoms at the end of life	Physician
Day 4-4	The role of the medical social worker	Medical social worker
Day 5		
Day 5-1	Radiotherapy	Physician
Day 5-2	Urinary symptoms at the end of life	Physician
Day 5-3	The role of the home physician	Physician
Day 5-4	Physical therapy approaches	Physical therapist
Day 6		
Day 6-1	Sedation for pain relief	Physician
Day 6-2	The role of the community pharmacist	Pharmacist
Day 6-3	Nerve block approaches	Physician
Day 6-4	The role of the pharmacist in the palliative care team	Pharmacist

**Table 2:** Pharmacists’ behavioral questionnaire questions regarding palliative care.

No	Contents of Question
Q1.	Are you confident in advising patients on opioid medications?
Q2.	Are you able to confidently make prescription suggestions and make inquiries to doctors regarding opioid medications?
Q3.	Do you understand the pain symptoms of patients with cancer and can you do something about them?
Q4.	Do you understand “the mental symptoms” of patients with cancer, and can you do something about them?
Q5.	Do you understand “the gastrointestinal symptoms” of patients with cancer, and can you do something about them?
Q6.	Do you understand “the respiratory symptoms” of patients with cancer, and can you do something about them?
Q7.	Do you understand “the urinary symptoms” of patients with cancer, and can you do something about them?
Q8.	Do you understand the role of “physicians” in palliative care and are you able to consult them when necessary?
Q9.	Do you understand the role of “nurses” in palliative care and are you able to consult them when necessary?
Q10.	Do you understand the role of “dietitians” in palliative care and are you able to consult them when necessary?
Q11.	Do you understand the role of “physical therapists” in palliative care and are you able to consult them when necessary?
Q12.	Do you understand the role of “medical social workers” in palliative care and are you able to consult them when necessary?

**Table 3:** Respondents’ characteristics.

		*n*	(%)
Age, years	20s	0	(0.0)
	30s	14	(14.6)
	40s	34	(35.4)
	50s	33	(34.4)
	60 years and above	15	(15.6)
Pharmacy experience, years	1–3	0	(0.0)
	3–5	1	(1.1)
	6–10	8	(8.3)
	11–20	37	(38.5)
	21 years or more	50	(52.1)
Workplace distribution	Community pharmacy	75	(78.1)
	Hospital	20	(20.8)
	Others	1	(1.1)
Place of residence	Fukuoka Prefecture	47	(49.0)
	Saga Prefecture	8	(8.3)
	Nagasaki Prefecture	3	(3.1)
	Kumamoto Prefecture	8	(8.3)
	Oita Prefecture	8	(8.3)
	Miyazaki Prefecture	4	(4.2)
	Kagoshima Prefecture	4	(4.2)
	Okinawa Prefecture	2	(2.1)
	Yamaguchi Prefecture	10	(10.4)
	Unknown	2	(2.1)

## Data Availability

The raw data supporting the conclusions of this study will be made available by the authors upon request.
